# Incorrect dosage of *IQSEC2,* a known intellectual disability and epilepsy gene, disrupts dendritic spine morphogenesis

**DOI:** 10.1038/tp.2017.81

**Published:** 2017-05-02

**Authors:** S J Hinze, M R Jackson, S Lie, L Jolly, M Field, S C Barry, R J Harvey, C Shoubridge

**Affiliations:** 1 Department of Paediatrics, Adelaide School of Medicine, University of Adelaide, Adelaide, SA, Australia; 2 Robinson Research Institute, University of Adelaide, Adelaide, SA, Australia; 3 The Genetic of Learning Disability Service, Waratah, NSW, Australia; 4 Department of Pharmacology, UCL School of Pharmacy, London, UK

## Abstract

There is considerable genetic and phenotypic heterogeneity associated with intellectual disability (ID), specific learning disabilities, attention-deficit hyperactivity disorder, autism and epilepsy. The intelligence quotient (IQ) motif and SEC7 domain containing protein 2 gene (*IQSEC2*) is located on the X-chromosome and harbors mutations that contribute to non-syndromic ID with and without early-onset seizure phenotypes in both sexes. Although IQ and Sec7 domain mutations lead to partial loss of *IQSEC2* enzymatic activity, the *in vivo* pathogenesis resulting from these mutations is not known. Here we reveal that *IQSEC2* has a key role in dendritic spine morphology. Partial loss-of-function mutations were modeled using a lentiviral short hairpin RNA (shRNA) approach, which achieved a 57% knockdown of *Iqsec2* expression in primary hippocampal cell cultures from mice. Investigating gross morphological parameters after 8 days of *in vitro* culture (8DIV) identified a 32% reduction in primary axon length, in contrast to a 27% and 31% increase in the number and complexity of dendrites protruding from the cell body, respectively. This increase in dendritic complexity and spread was carried through dendritic spine development, with a 34% increase in the number of protrusions per dendritic segment compared with controls at 15DIV. Although the number of dendritic spines had normalized by 21DIV, a reduction was noted in the number of immature spines. In contrast, when modeling increased dosage, overexpression of wild-type *IQSEC2* led to neurons with shorter axons that were more compact and displayed simpler dendritic branching. Disturbances to dendritic morphology due to knockdown of *Iqsec2* were recapitulated in neurons from *Iqsec2* knockout mice generated in our laboratory using CRISPR/Cas9 technology. These observations provide evidence of dosage sensitivity for *IQSEC2*, which normally escapes X-inactivation in females, and links these disturbances in expression to alterations in the morphology of developing neurons.

## Introduction

Cognition requires the formation and continued refinement of synaptic networks of neurons in the brain. Neurons display characteristic changes during development with outgrowth of axons and dendrites, and the subsequent establishment of synapses. Synapses are formed between axonal structures called boutons (the presynaptic compartment) and small protrusions on the dendrite called dendritic spines. These spines make up the postsynaptic component of most excitatory synapses in the mammalian brain. Dendrites of immature neurons have thin headless protrusions, filopodia, that lack the necessary postsynaptic components required for synapse function. With activity-dependent maturation of neurons, the number of filopodia rapidly decreases through pruning of these synaptic contacts with the simultaneous increase in the number of mature or bulbous spines.^1, 2, 3^ These spines can respond to extracellular signals and show morphological plasticity. Remodeling of synapses continues throughout life, with significant structural and functional synaptic plasticity during development in the late embryonic and early postnatal periods when synapse density is highest.^4^ The machinery driving the plasticity of dendritic spines involves remodeling of the actin cytoskeleton.^2, 5^ Actin dynamics and membrane trafficking is coordinated by the family of ADP ribosylation factors (ARFs), members of the Ras superfamily of small G proteins.^6^ Activation of ARFs is achieved by exchange of GDP for GTP nucleotide by a family of guanine nucleotide exchange factors (GEF) for the ARF family of GTP-binding proteins (ARFGEF). Intelligence quotient (IQ) motif and SEC7 domain containing Protein 2 (IQSEC2) is a GEF for ARF proteins^7^ and is localized to excitatory synapses as part of the NMDA receptor complex, via interactions with postsynaptic density proteins including disks large MAGUK scaffold protein 4 (DLG4) also known as PSD-95.^8^

Neurodevelopmental disorders are a large group of disorders that display disease onset during periods of maturation and development. Complex neuropsychiatric features of neurodevelopmental disorders include intellectual disability (ID), specific learning disabilities, attention-deficit hyperactivity disorder, autism and epilepsy. These disorders are linked to changes in development, involving alteration in neurogenesis, cell migration and neuronal connectivity that are responsible for cognitive deficits in later life. There is considerable genetic and phenotypic heterogeneity associated with these disorders. For example, monogenic causes on the X-chromosome alone include oligophrenin (*OPHNI*, a Rho GTPase activating protein; RhoGAP),^9^ Fragile X syndrome (*FMR1*),^10, 11^ p21 protein (Cdc42/Rac)-activated kinase 3 (*PAK3*),^12^ *ARHGEF6* (a Rho guanine exchange factor; RhoGEF)^13^ or *FGD1* (a RhoGEF)^14^ recently reviewed.^15^ Patients and animal models of neurodevelopmental disorders with early childhood onset show hallmarks of immature synaptic networks in the form of an increased filopodia-to-spine ratio and abnormal density of protrusions.^16, 17^

We first established the *IQSEC2* gene [NM_001111125] (MIM 300522) as a genetic cause of ID due to missense variants in X-linked familial cases.^18^ Since this report, high-throughput sequencing in ID or epilepsy cohorts has identified *de novo* loss-of-function *IQSEC2* mutations in males and increasingly in females.^19, 20, 21, 22, 23, 24, 25, 26, 27, 28, 29^ Abolition of enzymatic activity of IQSEC2 typically leads to a more severe phenotype including epileptic encephalopathy in both sexes. Recently, we have identified four male patients with ID and early speech disturbances with submicroscopic duplications at Xp11.22 containing the known ID genes *IQSEC2* and *KDM5C*^30^ indicating that dosage of IQSEC2 and/or KDM5C, or a combination thereof is important. In this study, we model the human disease state using knockdown and overexpression of *IQSEC2* and generate the first knockout mouse model for *Iqsec2* to investigate the impact and dosage of this gene on neuronal morphology, and in particular dendritic spine development.

## Materials and methods

### Animals

All animal procedures were approved by the Animal Ethics committee of the University of Adelaide, the SA Pathology Animal Ethics committee and the Animal Ethics committee of the Women’s and Children’s Hospital, Adelaide, SA, Australia. The pregnant Swiss dams were killed by cervical dislocation followed by decapitation of embryos.

### Construction of IQSEC2-containing vectors

The full-length IQSEC2 in pCAGGS incorporating an N-terminal FLAG-tag were a gift from Dr Randall Walikonis (University of Connecticut, Mansfield, CT, USA). The patient mutations and a previously reported dominant-negative IQSEC2^E849K^ mutation were generated by site-directed mutagenesis (Stratagene, San Diego, CA, USA) using pCAGGS^FLAG^IQSEC2: as a template. All clone preparations were verified by sequencing the entire *IQSEC2* complementary DNA (cDNA).

### Generation of shRNA triggers to knock down *Iqsec2*

Four shRNA sequences predicted to knock down mouse *Iqsec2* expression were designed using BLOCK-iT RNA designer (Invitrogen, Waltham, MA, USA). The sequences were chosen on the basis of species specificity and location within the messenger RNA (mRNA) transcript; shRA (5′-GGAAGTTCAGTCACCACATCT-3′) matches 3′ to the IQ-like domain in exon 4; shRB (5′-GCGACCTTCTAGTAGGCATCT-3′) and shRC (5′-TCATCATCTTTAATGCTCCTA-3′) are located in the Sec7 (exon 9) and the Pleckstrin Homology (exon 12) domains, respectively; All three triggers have at least 2 bp mismatched to the human sequence and matched identically to both NM_001114664 and NM_001005475 isoforms of mouse *Iqsec2*. Construction of shRNA clones was performed as outlined in Brown *et al.*,^31^ details supplied in Supplementary Methods. Endo-free maxi DNA preparations (Qiagen, Chadston, VIC, Australia) were used to generate large quantities of high-quality DNA for transfection and lentiviral packaging.

Lentivirus was prepared by transfecting three packaging vectors (VSV-g 3.75 μg DNA, Rev 6.25 μg DNA and gag/pol 7.5 μg DNA) together with 12.5 μg DNA of transfer vector plv-C-shRNA into packaging HEK293T cells. The media were changed after 18 h, and supernatant containing virus particles collected after a further 48 h. The supernatant was filtered through 0.45 μm filter, then centrifuged at 20 000 r.p.m. at 4 °C for 90 min. The pelleted lentiviral particles were resuspended in phosphate-buffered saline (PBS) in an appropriate volume and frozen in aliquots at −80 °C. The titer of each viral preparation was assessed using transduction rates of HEK293T cells (transduction efficiency determined using EGFP expression assayed by flow cytometry) with values ranging between 1.4 × 10^7^ and 1.1 × 10^9^ particles per milliliter.

### Neuronal cultures

The pimary hippocampal cultures were generated from embryonic day 17.5 (E17.5) Swiss mice. Unless stated otherwise, all cell culture reagents and supplements were sourced from Life Technologies (Waltham, MA, USA). The dissociated neurons were plated onto Biocoat poly-l-lysine coverslips (BD Biosciences, North Ryde, NSW, Australia) at 1.5 × 10^5^ cells per well in 24-well plates in Neurobasal-A medium supplemented with 10% fetal bovine serum (GIBCO, Waltham, MA, USA), 2% B27 and 2 mm glutamine. All the media were removed after 18 h and replaced with media excluding fetal bovine serum. The media were supplemented with 25 μm glutamate (Sigma, Sydney, NSW, Australia) for the first 6 days in culture. Routinely, half the volume of the media was refreshed every 2–3 days.

Before plating, primary hippocampal neurons were nucleofected with the Amaxa Mouse Neuron Nucleofector Kit (Lonza, Basel, Switzerland) using the protocol for mouse hippocampal neurons (program O-005). For transfection of shRNA constructs, a ratio of 1 μg DNA to 1 × 10^6^ cells was routinely used. For overexpression studies, a ratio of 12 μg DNA to 1 × 10^6^ cells was used, with a further 1–2 μg of pmaxGFP DNA (Lonza) in GFP-spiked groups. The individual cells were selected on the basis of GFP and FLAG (IQSEC2) expression. The cells were collected at days *in vitro* (DIV) 8, 15 and 21. The cells were fixed with 4% formaldehyde in PBS at room temperature for 1 h and stored at 4 °C until time of assay.

To generate larger cell numbers for RNA and protein analysis, cortical cultures (entire cortex including the hippocampus) were generated as stated for hippocampal cultures. The neurons were plated at 1.24 × 10^6^ cells per 60 mm^2^ dishes (BD Biosciences) coated with poly-l-lysine (Sigma), and 7 × 10^4^ cells per well in 24-well plates on a coverslip. Lentiviral transduction were performed 1 day post plating by the addition of 4.7 × 10^5^ particles of virus per 60 mm^2^ dish (multiplicity of infection at 0.4) at the first full media change (switching to fetal bovine serum-depleted media) on DIV1. The virus was subsequently removed 24 h later via complete media change. The neurons were harvested for RNA or protein at DIV8. The knockdown of *Iqsec2* was confirmed by immunoblot analysis and quantitative real-time reverse transcriptase PCR.

### Quantitative real-time reverse transcriptase PCR

Total RNA was extracted using Trizol (Life Technologies) and RNeasy Mini Kit (Qiagen) and treated with DNase I (Qiagen). We primed 2 μg of RNA with 1 μg of random hexamers and then subjected it to reverse transcription using the SuperScript III Reverse Transcriptase Kit (Invitrogen). The efficiency of transcription was tested by PCR using primers specific to the ubiquitously expressed *Actb* gene. Quantitative PCR was performed in triplicate using StepOnePlus Realtime PCR system (Applied Biosystems, Waltham, MA, USA) with products quantified by a relative standard curve prepared using adult mouse brain cDNA at neat, 10, 10^2^, 10^3^, 10^4^ dilution factors for each primer pair. The primer sequences are provided in Supplementary Methods.

### Protein analysis

For protein analysis, the cell lysates were prepared as previously described.^32^ The proteins were separated using 4–12% Tris-acetate SDS polyacrylamide gel electrophoresis in Tris-acetate running buffer, transferred to nitrocellulose membrane (PALL, Port Washington, NY, USA) for immunoblotting. The membranes were blocked 5% bovine serum albumin in Tris-buffered saline with 0.1% Tween-20 and incubated with appropriate primary and secondary antibodies (listed below) and developed with enhanced chemiluminesence method (GE Healthcare, Little Chalfont, UK).

### Immunofluorescence and microscopy

The cells were fixed using formaldehyde and permeabilized in 0.2% Triton in PBS for 5 min and blocked in 5% skim milk in Tris-buffered saline with 0.1% Tween-20. The primary and secondary antibodies were incubated in 1% skim milk in Tris-buffered saline with 0.1% Tween-20, with appropriate washing in Tris-buffered saline with 0.1% Tween-20 in between incubations. The cells were mounted using ProLong Gold Antifade with DAPI (Life Technologies). The neurons were washed twice with PBS after the secondary antibody and then counterstained with phalloidin (AlexaFluor Phalloidin-647; 1:1000, A22287, Life Technologies incubated in 1% bovine serum albumin in PBS) or CellTracker CM-DiI (C-7000, Molecular Probes, Waltham, MA, USA; at 1 μm CM-DiI in PBS for 20 min at 4 °C) before washing and mounting as above.

Fluorescence was viewed using the Axioplan2 microscope (Carl Zeiss, Jena, Germany) fitted with an HBO 100 lamp (Carl Zeiss). The images were captured using an Axiocam Mrm camera and Axio Vs40 v4.5.0.0 software (Axiovision, Carl Zeiss). The analysis of images was conducted using ImageJ software. For gross morphology, a straight line of defined length was drawn across the image with the center of the line aligned to the center of the cell soma. Using the Concentric Circle plugin on ImageJ software, the inner circle was set at 10 (and wholly contained within the cell soma) and the concentric circles arrayed at intervals of 24 μm. The number of times the neuron intercepted each odd concentric circle (excluding the circle within the soma) was counted manually. The numbers of biological replicates and individual neuron numbers counted for each measure are provided in the sample size and statistics.

To examine changes to dendritic spines, phalloidin-stained neurons expressing EGFP (that is, transduced or transfected) were selected and Z-stack images of neurons were acquired using a × 63 objective. The images were analyzed using Zeiss LSM 510 software. The segments of dendrite were manually traced and all protrusions stemming from the segment were added as regions of interest. The length of protrusions were measured by manually drawing a line from the base of the spine neck to the furthest point at the end of the spine. The protrusions were classified as filopodia if the length exceeded 3 μm, with shorter structures classed as spines. The spines were further subcategorized on the basis of length using the criteria from Vanderklish and Edelman,^33^ where stubby were <0.5 μm in length, mushroom were 0.5–1.25 μm in length and thin were 1.25–3.0 μm in length. For each neuron, a primary or secondary dendritic segment of 35 μm in length or greater was examined.

### Antibodies

For immunofluorescence studies, the primary antibodies used were mouse anti-FLAG (M2; 1:1000; Sigma F1804); chicken anti-MAP2 (1:1000; Millipore AB15452); mouse anti-tau (1:1000; Millipore MAB3420) and rabbit anti-β-tubulin-III (1:400; Sigma T2200). The secondary antibodies used for immunofluorescence were all sourced from Thermo Fisher (Waltham, MA, USA) and include; donkey anti-mouse IgG Alexa555 (1:2000 A31570); donkey anti-mouse IgG Alexa647 (1:1000 A31571) and donkey anti-rabbit IgG Alexa555 (1:2000 A31572). For immunoblotting, the primary antibodies were rabbit anti-hIQSEC2 (1:2000; details in Supplementary Methods), mouse β-actin (AC-74; 1:20 000; Sigma A2228) and rabbit anti-IQSEC3 (1:500; Abcam AB107853). Secondary antibodies from DAKO (Santa Clara, CA, USA) were goat anti-mouse HRP (1:2000 P0447) and goat anti-rabbit HRP (1:2000 P0448).

### Sample size and statistics

In neuronal cultures with shRNA knockdown, a minimum sample size of 22 was required to achieve a power of 80% with *P*=0.05 to detect a minimum difference in means of 0.5. A minimum number of three biological replicates were performed to ensure reproducible and robust changes. The images were captured and analysis undertaken with the investigator blinded to treatment or genotype. For the shRNA knockdown studies, each treatment group was tested at the three different time points in culture with the following sample numbers; morphological measures on neurons at 8DIV from shRL (*n*=47), shRA (*n*=39), shRB (*n*=34) and shRC (*n*=42) treatments. At 15DIV, the number of neurons/segments/protrusions (filopodia and spines) measured from shRL (*n*=18/62/528), shRA (*n*=15/53/813), shRB (*n*=12/35/244) and shRC (*n*=18/55/555). At 21DIV, the number of neurons/segments/protrusions (filopodia and spines) measured from shRL (*n*=4/14/222), shRA (*n*=7/15/241), shRB (*n*=5/12/155) and shRC (*n*=7/16/212). Overexpression studies were also tested at the two different time points in culture with the following sample numbers at 8DIV neurons from GFP (*n*=22), WT-IQSEC2 (*n*=36) and IQSEC2^E849K^ (*n*=22). At 21DIV, the number of neurons/segments/protrusions (filopodia and spines) measured from GFP (*n*=8/16/209), WT-IQSEC2 (*n*=6/10/91) and IQSEC2^E849K^ (*n*=5/13/111).

Statistical analysis on these measures was performed using SAS software, version 9.4 for Windows (SAS Institute, Cary, NC, USA). In cases of three groups, when normal distribution of data were assumed, a one-way analysis of variance was run to test whether there was an overall difference among the groups, and then planned comparisons were conducted to test difference between specific groups. Alternatively, when data were not distributed normally, a Kruskal–Wallis Test was run to test whether there was an overall difference among the groups, and then specific pairwise tests using the Wilcoxon rank-sum/Mann–Whitney *U*-test were conducted to test differences between specific groups. For comparisons of two treatments, independent *t*-tests and Wilcoxon rank-sum tests were used.

Cultures from the KO mice were performed across two biological replicates, each consisting of two pooled litters, generating 13 and 17 embryos, respectively. In total, 32 wild-type and 39 *Iqsec2* KO neurons were analyzed, respectively. Statistical analysis was performed using a Chi-squared test and complimentary Fisher's exact test of independence.

## Results

### Specific knockdown of *Iqsec2*

**Figure 1:**
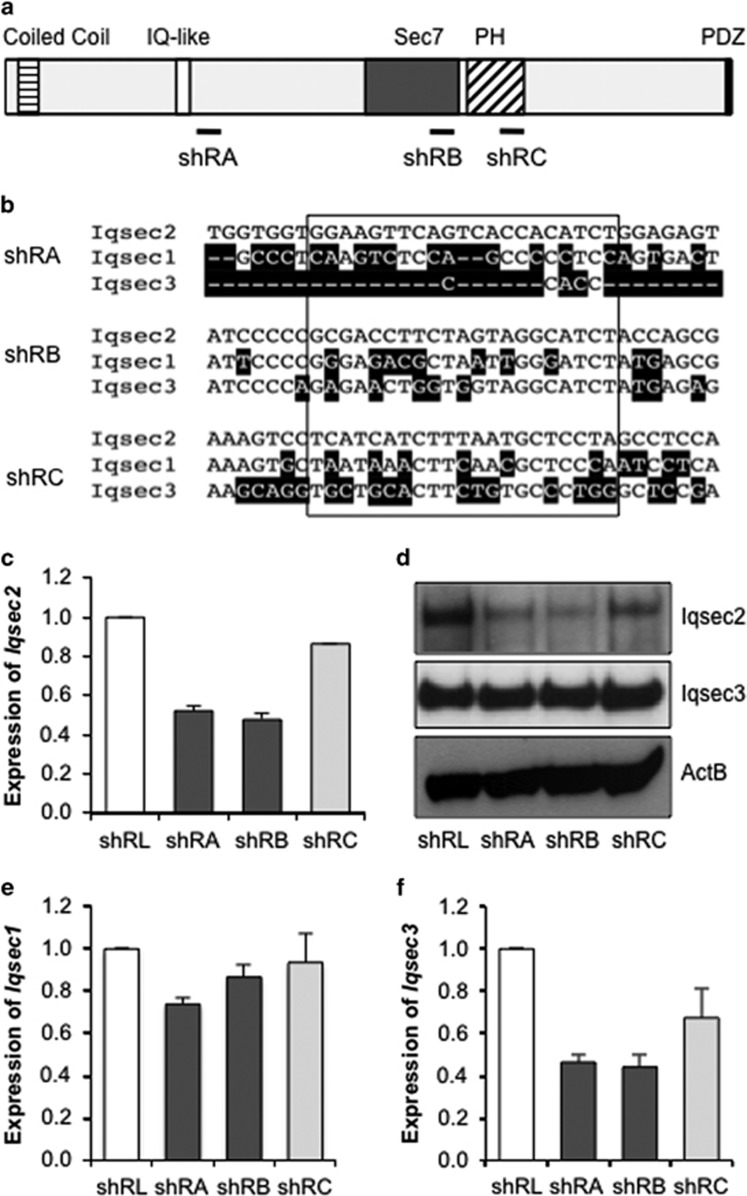
shRNA knockdown of Iqsec2. (**a**) Schematic of the IQSEC2 protein with domains highlighted; coiled coil (horizontal stripes), IQ-like (white), Sec7 (dark gray), Pleckstrin Homology (PH; diagonal stripes), PDZ binding motif (black). The locations of shRNA triggers are shown below relative to the domains. (**b**) Clustal W analysis of *Iqsec* family mRNA at shRNA target regions. *Iqsec1* shares 69% and *Iqsec3* shares 58% identity to *Iqsec2* at the mRNA level. The nucleotide bases that differ from *Iqsec2* are shaded black and the sequences targeted by the shRNA are boxed. (**c**) Expression of *Iqsec2* is reduced in primary neurons infected for 8 days with shRNA lentiviral vectors shRA and shRB compared with the shRL control. (**d**) Iqsec2 protein levels detected via western blot analysis of 20 μg protein from transduced primary neurons. Neurons infected with shRA and shRB vectors show reduced protein levels of Iqsec2 but no change in Iqsec3 protein levels. ActB was used as a loading control for each gel and a representative image is shown. (**e**) Expression of *Iqsec1* was not markedly reduced with either of these triggers against *Iqsec2*, however, we noted a decreased expression of *Iqsec3* (**f**). Relative expression of genes was normalized to the expression of the reference gene *ActB*. Relative gene expression for shRA- and shRB (light gray)-treated neurons is presented in comparison with shRL (white), which is defined as 1. IQSEC2, intelligence quotient motif and SEC7 domain containing protein 2 gene; mRNA, messenger RNA; shRNA, short hairpin RNA.

Nomenclature; Human: *IQSEC2* (gene or RNA)/IQSEC2 (protein) vs Mouse: *Iqsec2* / Iqsec2. To investigate the impact of *Iqsec2* knockdown on the gross morphology of neurons and the development of dendritic spines, we designed shRNA triggers interspersed across the full-length mRNA. The triggers shRA, shRB and shRC were to the open reading frame (Figure 1a), and specifically targeted mouse *Iqsec2* and not other family members *Iqsec1* and *Iqsec3* (Figure 1b). As a control, we used shRNA targeted to the luciferase gene, shRL. The shRNA triggers were cloned into a lentiviral transfer vector that permits expression of the shRNA together with EGFP. Lentiviral particles were generated and used to transduce cultured primary hippocampal and cortical neurons, resulting in 83–98% transduction rates (based on EGFP expression, data not shown).

Knockdown of *Iqsec2* expression in these cultures was achieved with shRA and shRB, which reduced *Iqsec2* mRNA expression by 52% and 48%, respectively (Figure 1c) and both lead to a marked decrease in the abundance of Iqsec2 protein (Figure 1d). By contrast, the shRC trigger resulted in a more modest knockdown of Iqsec2 at both the mRNA and protein levels when compared with the luciferase control (Figures 1c and d). These data were consistent across three separate neuronal harvest, culture and infection rounds (data not shown). At the mRNA level, *Iqsec1* is 69% homologous with *Iqsec2*, while *Iqsec3* is even lower at 58%. The shRNA triggers targeted regions of low homology (Figure 1b), therefore diminishing the likelihood of direct interference. As expected, the expression of *Iqsec1* in these cells was not markedly impacted by any of the triggers (Figure 1e). Interestingly, although the expression of *Iqsec3* was reduced at the mRNA level with the three specific *Iqsec2* triggers (Figure 1f), we did not see a reduction to the level of Iqsec3 protein in any case (Figure 1d). Taken together, these data indicated that the three triggers shRA, shRB and shRC were targeting *Iqsec2*.

### Knockdown of *Iqsec2* expression leads to altered morphology of developing hippocampal neurons

**Figure 2:**
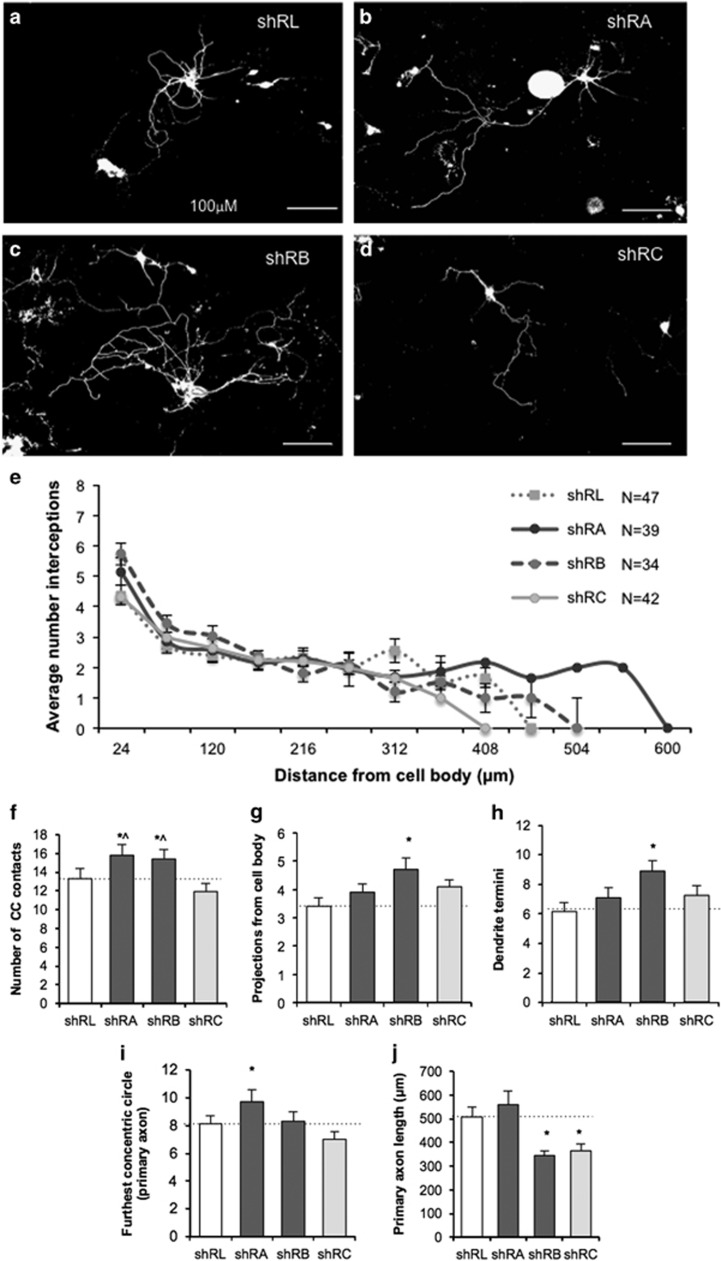
Knockdown of *Iqsec2* expression leads to altered morphology of developing hippocampal neurons. Hippocampal neurons were examined after 8 days in culture (8DIV) for gross morphological measures following knockdown of endogenous *Iqsec2*. In the top panel, pictomicrographs of hippocampal neurons transfected with (**a**–**d**) control shRL, shRA, shRB and shRC, respectively. (**e**) Scholl analysis of neuron interceptions on concentric circles at distance from the cell soma show broader spread for neurons with knockdown of Iqsec2; (control shRL pale gray dotted line; shRA solid dark gray line, shRB dark gray dashed line; shRC in pale gray solid line). Morphological measures include the number of times the neuron intercepted the concentric circles (**f**), the number of projections from the cell body (**g**), number of dendrite termini (**h**), the furthest concentric circle reached by the primary axon (**i**) and the length of the primary axon (**j**) for neurons transfected with shRNA vectors to reduce Iqsec2 expression (control shRL in white bars; shRA and shRB analysis in dark gray bars; shRC in pale gray bars). Data represent mean+s.e.m., from shRL (*n*=47), shRA (*n*=39), shRB (*n*=34) and shRC (*n*=42) treated neurons from three biological replicates. Scale bar, 100 μm. Significance *P<0.05 vs shRL and ^*P*<0.05 vs shRC. DIV, days *in vitro*; IQSEC2, intelligence quotient motif and SEC7 domain containing protein 2 gene; shRNA, short hairpin RNA.

To assess the effect of *Iqsec2* knockdown, dissociated neurons were isolated from E17.5 wild-type mice and nucleofected with shRNA constructs which co-express EGFP, and analyzed after 8 days of *in vitro* culture (8DIV). Knockdown of *Iqsec2* resulted in neurons with greater neurite complexity compared with the control shRL (Figures 2a–d). To quantify these differences, we performed a modified Sholl analysis: concentric circles were overlaid on imaged neurons, centered over the cell soma and the average number of neuron interceptions at increasing radii scored. Neurons nucleofected with shRA and shRB have extensions further from cell soma than either control shRL or shRC (Figure 2e).

We assessed the complexity of the neurons using multiple metrics. The number of times the neuron intercepted a concentric circle was significantly increased in the knockdown of *Iqsec2* with shRA and shRB when compared with shRL control or the more modest reduction in *Iqsec2* expression of the shRC treatment (Figure 2f). The number of projections from the cell body (Figure 2g), and the branching of the neurites measured by the number of termini (Figure 2h) was both significantly increased in shRB compared with the shRL control. Interestingly, both these measures were also higher in the other two knockdown triggers of shRA or shRC, but the changes were not significant compared with shRL. The spread of the neurite processes captured by measuring the furthest concentric circle reached by the primary axon was significantly increased for the knockdown of *Iqsec2* with shRA compared with all other treatments (Figure 2i). This may be in part due to the significantly shorter length of the primary axon measured for both shRB and shRC compared with shRL (Figure 2j). Interestingly, when considering the impact of dosage of *Iqsec2* on these measures, the shRC trigger with only a very modest reduction to Iqsec2 expression (Figure 1) provides a good control to the more robust knockdown achieved with shRA or shRB. Except for primary axon length (Figure 2j), the measures for neurons with shRC treatment were not significantly different to neurons treated with shRLuc control. This was in comparison with the significantly increased values across the various morphological measures for neurons treated with shRA or shRB (or both; Figures 2f–i).

### Knockdown of *Iqsec2* expression increases the density of dendritic spines

**Figure 3:**
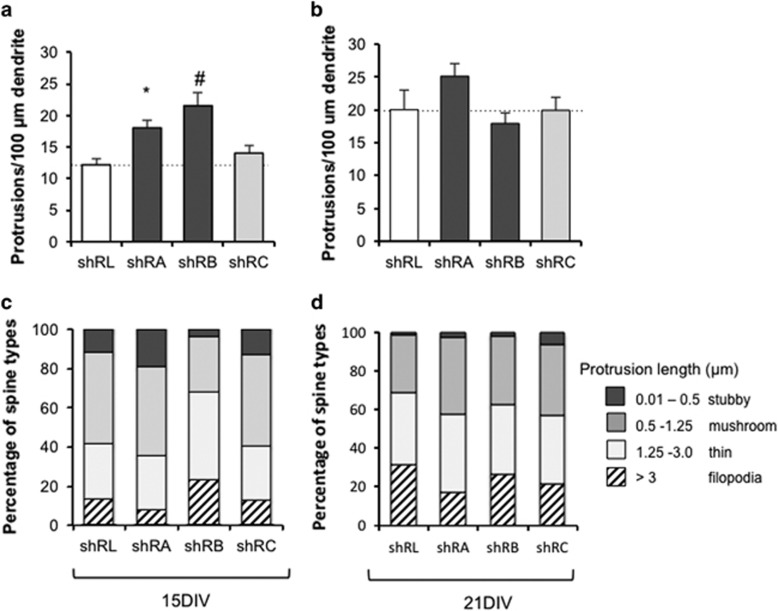
Knockdown of *Iqsec2* expression increases the density of protrusions in hippocampal neurons. The density of protrusions on dendrites in hippocampal neurons transfected with shRNA vectors to reduce *Iqsec2* expression were measured after 15 (15DIV; **a**) and 21 days in culture (21DIV; **b**); control shRL (white bars), shRA and shRB (dark gray bars), shRC (pale gray bars). At 15DIV (**c**) and 21DIV (**d**), the percentage of the total protrusions were divided into categories of diminishing size, as indicated on the right hand side of the figure, with stubby spines (dark gray), mushroom spines (medium gray), thin spines (pale gray) and filopodia in hatched bars. Data represent mean+s.e.m. At 15DIV, the number of neurons/segments/protrusions (filopodia and spines) measured from shRL (*n*=18/62/528), shRA (*n*=15/53/813), shRB (*n*=12/35/244) and shRC (*n*=18/55/555). At 21DIV, the number of neurons/segments/protrusions (filopodia and spines) measured from shRL (*n*=4/14/222), shRA (*n*=7/15/241), shRB (*n*=5/12/155) and shRC (*n*=7/16/212). Measures for each group from three biological replicates. Significance *P<0.05 or ^#^*P*<0.001 compared with shRL control. DIV, days *in vitro*; IQSEC2, intelligence quotient motif and SEC7 domain containing protein 2 gene; shRNA, short hairpin RNA.

When dissociated hippocampal neurons are cultured, the number and maturity of dendritic spines increases steadily, peaking at 3 weeks in culture. Across this time, the proportion of filopodia declines concordant with a rise in the number of smaller mature spines. By 3 weeks in culture, dendritic spines have the characteristics of synaptic terminals and are similar to those seen *in vivo*.^34^ To investigate the impact of knockdown of *Iqsec2* on spine morphology, we analyzed the density and type of dendritic protrusions of hippocampal neurons. After 15 days in culture (15DIV), *Iqsec2* knockdown using the shRA and shRB triggers resulted in significant increases of 35% and 44%, respectively in the density of protrusions compared with controls (Figure 3a). However, when the dosage of *Iqsec2* expression was not markedly altered in the short hairpin trigger that resulted in less effective knockdown of *Iqsec2,* there was only a very modest and not significant increase of 13% in protrusion density compared with the controls. The increase in protrusion density compared with controls was diminished at 21 days in culture (21DIV; Figure 3b). To examine changes to the maturity of the spines on neurons with altered *Iqsec2* expression, each of the protrusions were classified as filopodia (if the length exceeded 3 μm) or spines (if length less than 3 μm). The spines were further subclassified on the basis of length using the criteria from Vanderklish and Edelman^33^ as stubby (< 0.5 μm in length), mushroom (0.5–1.25 μm in length) and thin (1.25–3.0 μm in length). At 15DIV, we did not observe a consistent change to the proportion of spines to filopodia between the shRL controls and Iqsec2 knockdown triggers (Figure 3c). In neurons allowed to continue development in culture until 21DIV, we identified a modest reduction in the proportion of longer immature filopodia in neurons nucleofected with *Iqsec2* knockdown triggers compared with shRL controls, particularly shRA (Figure 3d). The concomitant increase in dendritic spines was observed across all subclassifications of spine types.

### Dosage of *IQSEC2* alters the morphology of developing neurons and increases the maturity of dendritic spines

To investigate further the impact of dosage of *Iqsec2* expression on the development of neurons, overexpression of wild-type *IQSEC2* was compared with GFP transfected controls. The *IQSEC2* cDNA is considerable in size (IQSEC2: NM_0011111125 is 4464 bp in the pCAGGS vector backbone of over 4790 bp) and as such the impact of the introduction of such a large construct to neurons in culture on the morphology independent to the specific impact of the overexpressed protein was unclear. To attempt to control for this, we used a dominant-negative mutation E849K that changes the invariant E residue of the Sec7 domain and lacks Arf-GEF enzymatic activity.^35^ Our previous use of this variant indicates a significant reduction (> sixfold) to the Arf-GEF enzymatic activity *in vitro*.^18^ Hence, when analyzing the data, we were principally looking for changes in morphology observed with wild-type *IQSEC2* in comparison with both the IQSEC2^E849K^ mutant and GFP controls.

**Figure 4:**
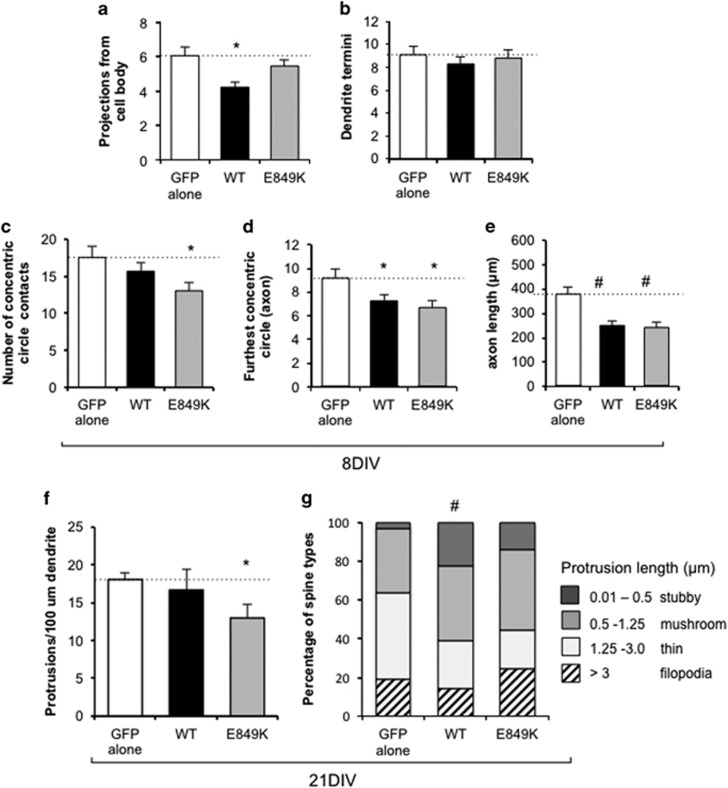
Dosage of *IQSEC2* alters the morphology of developing neurons and increases the maturity of dendritic spines. Morphological measures examined after 8 days in culture (8DIV) include the number of projections from the cell body (**a**), number of dendrite termini (**b**), the number of times the neuron intercepted the concentric circles (**c**), the furthest concentric circle reached by the primary axon (**d**) and the length of the primary axon (**e**) for hippocampal neurons transfected with overexpression of *IQSEC2* wild-type. At 21 days in culture (21DIV), the density of protrusions on dendrites in hippocampal neurons with overexpression of *IQSEC2* (**f**) was measured. Control GFP alone (white bars), wild-type *IQSEC2* (WT; black bars), IQSEC2^E849K^ dominant-negative mutation (E849K; light gray bars). The percentage of the total protrusions at 21DIV were divided into categories of diminishing size, as indicated on the right hand side of the figure, with stubby spines (dark gray), mushroom spines (medium gray), thin spines (pale gray) and filopodia in hatched bars (**g**). Data represents mean+s.e.m. 8DIV neurons from GFP (*n*=22), WT-IQSEC2 (*n*=36) and IQSEC2^E849K^ (*n*=22) for each group from three biological replicates. At 21DIV, the number of neurons/segments/protrusions (filopodia and spines) measured were from GFP (*n*=8/16/209), WT-IQSEC2 (*n*=6/10/91) and IQSEC2^E849K^ (*n*=5/13/111). Significance *P<0.05 or ^#^*P*<0.001 compared with GFP alone control. DIV, days *in vitro*; GFP, green fluorescent protein; IQSEC2, intelligence quotient motif and SEC7 domain containing protein 2 gene.

At the early time points in culture, overexpression of wild-type *IQSEC2* significantly reduced the number of projections from the cell body (Figure 4a) and resulted in a subtle reduction in the number of neurite termini (Figure 4b) compared with both GFP alone and the IQSEC2^E849K^ mutant neurons. This was in contrast to the increases in both these measures when the activity of *Iqsec2* was knocked down. However, in agreement with the data from the knockdown of *Iqsec2*, overexpression of wild-type *IQSEC2* resulted in a reduced number of times the neuron intercepted a concentric circle (Figure 4c) and a significant reduction in both the furthest concentric circle reached by the primary axon and the length of the primary axon (Figures 4d and e) when compared with GFP alone. However, each of these measures was also reduced in the IQSEC2^E849K^ mutant treated neurons when compared with GFP alone. We cannot rule out that the delivery and expression from the plasmid itself is contributing to a reduction in these morphological measures. As we were delivering full-length IQSEC2, this outcome raises the possibility that another aspect of IQSEC2 function that does not relate to the Sec7 enzyme activity (for example, IQ domain or PDZ domain interactions) may be impacting these morphological measures. When considered together with the data from the knockdown of Iqsec2, we demonstrate that the number of projections from the cell body and neurite termini are both impacted in a dosage-dependent manner with discordant outcomes depending on knocking down *Iqsec2* or overexpressing *IQSEC2*. However, the measures highlighting development and growth of the axon were reduced with any alteration to *Iqsec2/IQSEC2* expression.

At longer time points in culture, neurons overexpressing wild-type *IQSEC2* displayed a slight decrease in the density of projections on dendrites (12% at 15DIV and 8% at 21DIV), with a significant reduction (22% at 21DIV) for the IQSEC2^E849K^ mutant compared with GFP alone (Figure 4f). When we examined the maturity of the spines on neurons with wild-type *IQSEC2* overexpression, there was an increase in the proportion of stubby (<0.5 μm in length) and mushroom (0.5–1.25 μm in length) subclasses of spines, at the expense of thin spines (1.25–3.0 μm in length) and immature filopodia (length exceeded 3 μm; Figure 4g). This was significant when either analyzed as odds ratio for being in a shorter-length category or if protrusion length was log transformed and estimates back transformed. There was no significance difference in the proportion of spine subclass in the IQSEC2^E849K^ mutant treatment group compared with either GFP alone or wild-type *IQSEC2* overexpression (Figure 4g).

### Neurons from *Iqsec2* knockout mice exhibit gross morphological disturbances during early growth and development

**Figure 5:**
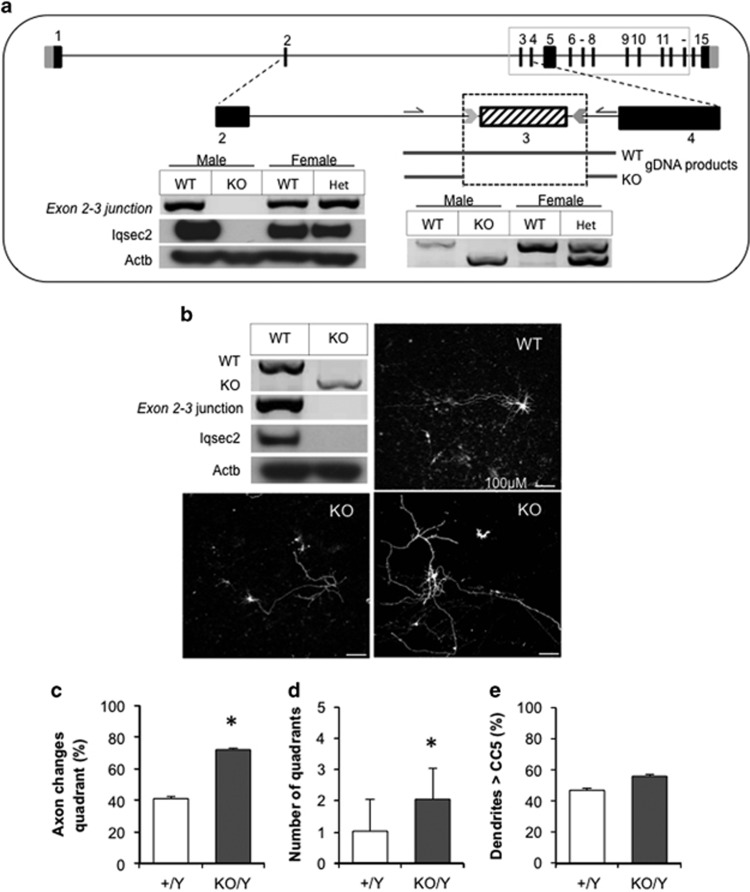
Cortical neurons from *Iqsec2* knockout (KO) mice demonstrate early gross morphological changes when compared with their wild-type (WT) counterparts. (**a**) The exon–intron structure of the longest isoform of *IQSEC2* gene [NM_0011111125] has 15 exons, with the ATG, open reading frame and stop codon positions in black and 5′ and 3′ untranslated regions in light gray. The sequence of exon 3 to exon 13 are identical (boxed with solid gray line). The region targeted by CRISPR/Cas9 is highlighted below with knockout of exon 3 shown at the gDNA level (below dotted box) and the PCR amplicons in the panel on the left depicts the absent *Iqsec2* mRNA expression across the exon 2–3 junction and absent Iqsec2 protein abundance in hemizygous males (KO/Y) compared with wild-type (+/Y, WT) controls. Heterozygous females (KO/+) and their wild-type controls (+/+) are shown for reference, as heterozygous females were used to generate subsequent experimental embryos. (**b**) The absence of PCR amplicons and Iqsec2 protein abundance was confirmed in cortical neurons extracted from hemizygous (KO) males compared with wild-type (WT) controls. Representative pictomicrographs of cortical neurons spiked with 0.5 μg pmaxGFP from WT and KO males. Scale bar, 100 μm. (**c**–**e**) Neurons were examined after 8 days in culture (8DIV) with measures for WT (white bars) or KO neurons (dark gray bars) including the proportion of primary axons which change quadrant (1 quadrant=45 degrees; **c**), number of quadrants changed (**d**) and proportion of neurons where dendrites project further than concentric circle 5 (CC5; **e**). Data represent mean+s.e.m., from neurons from Wt (*n*=32) and *Iqsec2* KO (*n*=39), from cultures of two biological replicates consisting of two pooled litters, generating 13 and 17 embryos, respectively. *P<0.05 indicates significant difference between WT and KO neurons. DIV, days *in vitro*; gDNA, genomic DNA; IQSEC2, intelligence quotient motif and SEC7 domain containing protein 2 gene; mRNA, messenger RNA.

Aspects of the disturbances to gross morphology of hippocampal neurons due to knockdown of *Iqsec2 in vitro* were recapitulated in cortical neurons harvested from *Iqsec2* knockout mice (Figure 5). Knockout of *Iqsec2* expression was achieved by targeting CRISPR/Cas9 removal of exon 3 in a C57Bl/6 background (Figure 5a). Although the behavioral and phenotypic characterization of this knockout mouse is ongoing (MRJ, personal communication) and outside the scope of the current report, we have confirmed transmission of the exon 3 removal and subsequent loss of *Iqsec2* mRNA expression and Iqsec2 protein in hemizygous male knockout offspring (Figure 5a). Heterozygous *Iqsec2* knockout females were time-mated with wild-type males and cortical neurons extracted from individual embryos at E17.5 days post coitum (d.p.c.). Concomitant genotyping from tail material of each embryo allowed us to pool genotypically relevant neurons before culturing cells as outlined for shRNA cultures.

At 7DIV, the cells were transfected with a 0.5 μg spike of pmaxGFP to visualize individual neurons and cells collected and fixed 24 h later (8DIV). *Iqsec2* knockout neurons were more disorganized in their arrangement when compared with neurons from wild-type littermates (Figure 5b). Unlike the knockdown or overexpression neurons, there were no significant disturbances to the number of projections from the cell body or branching of the neurites (number of termini) in the knockout neurons compared with neurons from wild-type littermates (data not shown). We cannot rule out that use of cortical cultures in the case of KO neurons as opposed to hippocampal neurons in culture for the shRNA knockdown experiments may contribute to this discrepancy. In keeping with the data from the shRNA knockdown, we did note a trend towards longer dendritic processes from the cell body in *Iqsec2* knockout neurons (56%) when compared with neurons extracted from their wild-type littermates (47%). However, this did not reach significance (Figure 5e). Similar to previous manipulation of *Iqsec2/IQSEC2*, the primary axon was sensitive to complete loss of *Iqsec2*. Although the majority of primary axons in wild-type neurons (59%) followed a defined course, or displayed a limited change in direction (under 45 degrees=1 quadrant), 72% of primary axons in *Iqsec2* knockout neurons altered their course on average by more than 90 degrees (two quadrants; Figures 5c and d; Chi-squared test *P*=0.0082 and *P*=0074, respectively).

## Discussion

We provide experimental support for a role of Iqsec2 in modulating the morphology of developing neurons, including the development of dendritic spines. We show increased complexity and spread of neurite processes in neurons with knockdown of *Iqsec2,* whereas by contrast, increased expression of *IQSEC2* leads to neurons with shorter axons that are more compact and display simpler dendritic branching. Observations in neurons obtained from hemizygous *Iqsec2* knockout mice support the impact of Iqsec2 on neuronal morphology and, in particular, highlight that disturbances in the expression levels of *Iqsec2* disrupt normal axonal development. Taken together, the data from our investigations provide evidence of dosage sensitivity for this X-chromosome gene that normally escapes X-inactivation in humans (but not in mice) and links for, to the best of our knowledge, the first time these disturbances in expression with alterations in the morphology of developing neurons.

IQSEC2 catalyzes exchange of GDP for GTP in some members of the ARF protein superfamily. The identification of variants in families with X-linked intellectual disability that clustered around the Sec7 and IQ-like domains and resulted in reduced enzymatic activity, provided strong evidence that missense mutations in *IQSEC2* cause a neurocognitive disability.^18^ Affected male patients with these variants displayed non-syndromic ID, with variable penetrance of other comorbidities such as autistic spectrum disorders and seizures. The females in these families were generally well and did not display a consistent phenotype, but there was variable penetrance of carrier females being less academically able compared with non-carrier siblings.^18^ Initially, it was unclear whether loss-of-function variants in *IQSEC2* in males would be compatible with life.

What was known about IQSEC2, also referred to as BRAG1, was that levels of expression increased with postnatal development with the strongest expression in the brain, particularly in the cerebellum, cortex and hippocampus, including the dentate gyrus.^7, 8^ Moreover, Iqsec2 is localized to excitatory synapses as part of the NMDA receptor complex, via interactions with postsynaptic density proteins DLG1, DLG2, DLG3 and DLG4.^8^ *Iqsec2* mRNA and protein have been identified at dendrites suggesting that *Iqsec2* mRNA might be locally translated in an activity-dependent manner thereby contributing to synaptic plasticity. This body of evidence underscores the role for IQSEC2 in the fundamental process of dendritic development. Our study aimed to examine the impact of *Iqsec2* on this process, and using a knockdown approach we have demonstrated for the first time that reduced expression of *Iqsec2* resulted in increased neurite processes in hippocampal neurons at early time points in culture, with a transient increase in the density of protrusions at 15 days *in vitro* and a propensity for shorter more mature dendritic spines after 21 days. The utility of these types of investigations is highlighted by the elegant original study by Govek *et al.*^9^ in which RNA interference was used to model naturally occurring *OPHN1* mutations in CA1 neurons, with downregulation of this gene resulting in significantly shortened dendritic spines, shown to be related to RhoA/Rho-kinase signaling pathway. More broadly, patients and animal models of neurodevelopmental disorders with early childhood onset show hallmarks of immature synaptic networks, in the form of an increased filopodia-to-spine ratio or abnormal density of protrusions.^16, 17^

In contrast to the deficits to *IQSEC2* function, we have recently reported four male patients with ID with early speech disturbances with submicroscopic duplications at Xp11.22 containing the known ID genes *IQSEC2*, *KDM5C* and *TSPYL2*.^30^ These data indicated that *IQSEC2* may be a dosage-sensitive gene. Hence, we wanted to investigate the impact of dosage of *IQSEC2* in our neuronal culture model. Overexpression of wild-type *IQSEC2* resulted in a significant decrease in the number of neurite processes at the early stages of neuron culture, the opposite trend to that observed in our knockdown studies. We also observed a significant shift toward shorter, more mature dendritic spines with overexpression of wild-type *IQSEC2* for 3 weeks in culture. These observations provide additional support for dosage sensitivity of *IQSEC2*. In support, a recent study demonstrated that IQSEC2 modulates AMPA receptor-mediated synaptic transmission in a bidirectional manner: with *IQSEC2* knockdown reducing synaptic transmission, but overexpression enhancing transmission.^36^ Taken together, these observations provide additional support for dosage sensitivity of *IQSEC2*.

As the first variants reported for *IQSEC2*, high-throughput sequencing in ID and epilepsy cohorts has increasingly identified *de novo* loss-of-function *IQSEC2* mutations in males and increasingly in females.^19, 20, 21, 22, 23, 24, 25, 26, 27, 28, 29^ Complete abolition of enzymatic activity does not lead to lethality, but instead leads to a severe phenotype including epileptic encephalopathy in both males and females. The novel *Iqsec2* knockout mouse we have generated is viable during postnatal life (both hemizygous males and herterozygous females) and provides a valuable resource to better interrogate the impact of Iqsec2 dysfunction. Harnessing neurons from this knockout mouse in culture, we were able to re-capitulate some of the findings from our shRNA knockdown studies, in particular of the gross morphological studies seen at early stages of neuronal development. Interestingly, we noted discrepancies in several morphological measures in the KO neurons when compared with the shRNA knockdown studies. We cannot exclude at least some contribution due to the technical nature of the different experiments (that is, neurons cultured from different mouse strains and hippocampal vs cortical neuronal cultures). However, a less pedestrian consideration may be that the differences we see between the two approaches represent contributors to the spectrum of phenotypic outcomes that we see in patients with various mutation types in *IQSEC2*. This perhaps reflects that a partial loss of function of a particular gene may have a very different impact compared with a complete loss of function in the same setting. Indeed, this has been noted for many non-syndromic ID genes. In contrast, one of the most interesting findings evident across each of the manipulations we undertook in this study, was the negative impact on axon length, morphology and directionality when expression of *Iqsec2/IQSEC2* was disturbed. When another ARFGEF (EFA6A) is ablated, a decrease in spine formation in noted.^37^ Investigating the role of ARF6 in conjunction with EFA6A indicates that ARF6 knockdown decreases the conversion of filopodia to spines and stability of early spines.^37^ The IQSEC family display a unique domain structure compared with all other ArfGEFs, each containing a coiled-coiled domain (CC), an IQ-like domain (IQ), the catalytic SEC7 domain (Sec7) and a Pleckstrin Homology domain. We speculate that compromise of functions other than just the Sec7 catalytic activity of IQSEC2 might be contributing to the disturbed morphology of the neurons following altered levels of *Iqsec2/IQSEC2*. The preferential accumulation of IQSEC2 in the tips of dendritic spines has been shown to be altered with the removal of last 74 amino acids, implicating the C terminus of IQSEC2,^38^ which contains a PDZ domain binding motif. A recent study strengthens this finding, with *IQSEC2* expression enhancing synaptic transmission independently of Arf-GEF or neuronal activity, but dependent on IQSEC2 C-terminal interactions.^36^ In regard to the other IQSEC family members, there is no known disease-causing mutations in either IQSEC1 or IQSEC3 currently described. The role of these proteins on synaptic function, particularly for the other predominately brain expressed member, IQSEC3, is growing and has recently been reviewed.^39^

The *Iqsec2* knockout mouse reported here has the potential to provide a platform to address broader questions relating to functional aspects of this interesting gene. For example, these neurons will provide a potential means to establish the role that *Iqsec2* has specifically in modulating activity-dependent processes downstream of signaling via the NMDA receptor. In addition, the deficits due to specific patient mutations in *IQSEC2* may be modeled using rescue experiments, to assist in determining the pathogenicity of variants of unknown significance. Moreover, treatments to improve aspects of the development of neurons when IQSEC2 function is deficient may be modeled. In conclusion, our study has utilized a range of approaches to knockdown, overexpress or completely knockout *Iqsec2/IQSEC2* expression in primary hippocampal neurons. We have demonstrated a role for this known ID and seizure gene on the morphology of the neuron and development of dendritic spines, including a negative impact on the growth of neuronal axons. These observations provide support for dosage sensitivity for *IQSEC2*, which normally escapes X-inactivation in humans and for the first time links these disturbances in expression with specific alterations to the morphology of developing neurons.
