# Supplementary material for: Incorrect dosage of IQSEC2, a known intellectual disability and epilepsy gene, disrupts dendritic spine morphogenesis
Source: Transl Psychiatry. 2017 May 2;7(5):e1110–. doi: 10.1038/tp.2017.81 (PMC5534949; doi:10.1038/tp.2017.81)
Supplement: Supplementary Information [file tp201781x1.docx]

**Supplementary methods**

**Generation of shRNA constructs**

In brief, shRNAs were cloned using a three-step PCR approach. PCR 1 used attB1-F primer and shRNA-R primer specific to each shRNA. Primer sequences are available upon request. The template was pBS-tH1 with the resulting PCR fragment containing tH1 promoter and sense portion of shRNA including stem loop. PCR2 used shRNA-F primer specific to each shRNA and attB1-R primer. No template was needed as primers overlap. PCR2 fragment contains stem loop and anti-sense portion of shRNA. PCR 3 used attB1-F and attB1-R primers and column-purified PCR1 and PCR2 products as template. Final PCR3 product contains tH1 promoter, sense shRNA, stem loop and anti-sense shRNA flanked by attB sites. Final PCR3 product size was 349 bp. PCR conditions for all three reactions were 94°C for 2 min, 35 cycles of 94°C for 45 sec, 50°C for 45 sec, 72°C for 45 sec, then a single 72°C step for 5 min. Once generated, the PCR3 fragment for each shRNA was subsequently cloned into plv-C-716 vector (lentivirus backbone with Gateway cassette upstream of hEF1α promoter driving dGFP) via Gateway cloning (Invitrogen). All constructs were sequenced to confirm tH1 and shRNA sequence.

**Primers for real-time qPCR**

Iqsec2-F 5’- CTC CAA ACA GGT AAA GTC TCT GGC TG-3’, Iqsec2-R 5’- GAG CTG TCC TCT TGC TGT TCC T-3’ (product size 145 bp), Iqsec1-F 5’-ACG TGT CCC AGG TGC AGA AGG TTG-3’, Iqsec1-R 5’-TGG GGT TTG TTG GGG TCA GGA A-3’ (product size 154 bp), Iqsec3-F 5’-GGT GCA GAC ATC CCC AGA GAA CT-3’, Iqsec3-R 5’-GAA GAG CCG GCT ACA GCA GAC CAG-3’ (product size 177 bp), ActB-F 5’-GAT ATC GCT GCG CTG GTC GTC-3’ and ActB-R 5’-TCT CTT GCT CTG GGC CTC GTC AC-3’ (product size 177 bp) (Geneworks, Adelaide).

**Generation of a polyclonal antibody against IQSEC2**

We generated polyclonal antibodies against the longer IQSEC2 isoform using a peptide (GPRPPRERGQLSRGASRSSSP) against p.195-215 of NP_001104595. Synthetic peptides (Mimitopes) were conjugated to diphtheria toxoid and used to immunize NZ white rabbits (6 injections across 10 weeks) (Flinders Partners Antibody Facility, Flinders University of South Australia). Serum was collected two weeks after the sixth inoculation and antibodies were then purified using HiTrap NHS Activated Sepharose Columns (GE Healthcare) via FPLC.
